# A comparative analysis of Denosumab and Zoledronic acid effects on bone metabolism and bone mineral density in individuals with osteoporotic vertebral compression fractures

**DOI:** 10.5937/jomb0-52084

**Published:** 2025-03-21

**Authors:** Shouda Gao, Lijun Zhang, Liyan Liu, Jiayin Meng

**Affiliations:** 1 The Fifth People's Hospital of Jinan, Department of Orthopedics, Jinan, China; 2 The Fifth People's Hospital of Jinan, Department of Nephropathy, Jinan, China; 3 Jinan Second People's Hospital, Department of Pharmaceutical, Jinan, China

**Keywords:** osteoporotic vertebral compression fractures, serum bone alkaline phosphatase (BAP), total procollagen type 1 amino-terminal propeptide (TP1NP), bone gamma carboxy-glutamic acid containing proteins (BGP), denosumab, zoledronic acid, Bone mineral density, clinical safety, economic benefits, osteoporotske kompresione frakture pršljenova, serumska alkalna fosfataza kostiju (BAP), ukupni prokolagen tipa 1 aminoterminalni propeptid (TP1NP), proteini koji sadrže gama-karboksiglutaminsku kiselinu (BGP), denosumab, zoledronska kiselina, gustina minerala u kostima, klinička bezbednost i ekonomska korist

## Abstract

**Background:**

This study aimed to compare the efficacy of Denosumab (DEN) versus Zoledronic acid (ZOL) in treating patients with osteoporotic vertebral compression fractures (OVCF) after percutaneous kyphoplasty (PKP).

**Methods:**

In this study, 128 OVCF patients who underwent PKP in our hospital from June 2019 to August 2022 were selected and randomized into a DEN group treated with DEN and a ZOL group treated with ZOL. Bone mineral density (BMD), bone metabolism, pain, and lumbar mobility were compared between the two groups before and after treatment, and the anterior vertebral height and local kyphotic angle were measured. Finally, the one-year refracture rate and treatment cost were counted.

**Results:**

The two groups showed no notable difference in pain, lumbar mobility, anterior vertebral height, and local kyphotic angle after treatment (P>0.050). BMD was higher in the DEN group at 6 and 12 months after treatment, while b isomer of C-terminal telopeptide of type I collagen (b-CTX) was lower (P<0.050). No statistical inter-group difference was identified in the one-year re-fracture rate (P>0.050), while the total treatment cost was lower in the DEN group compared with ZOL group (P<0.050).

**Conclusions:**

DEN and ZOL effectively improve the prognosis of OVCF patients after PKP, but DEN can more significantly improve BMD and bone metabolism with higher economic benefits.

## Abstract (sr)

**Uvod:**

Cilj ove studije bio je da uporedi efikasnost denosumaba (DEN) i zoledronske kiseline (ZOL) u lečenju pacijenata sa osteoporotskim kompresionim frakturama pršljenova (OVCF) nakon perkutane kifoplastike (PKP).

**Metode:**

U ovoj studiji, odabrano je i nasumično podeljeno u grupu koja je lečena denosumabom (DEN) i grupu koja je lečena zoledronskom kiselinom (ZOL) 128 pacijenata sa osteoporotskim kompresionim frakturama pršljenova (OVCF) koji su prošli perkutanu kifoplastiku (PKP) u našoj bolnici od juna 2019. do avgusta 2022. godine. Gustina minerala u kostima (BMD), metabolizam kostiju, bol i pokretljivost lumbalne kičme su upoređivani između dve grupe pre i posle lečenja, a mereni su visina prednje strane pršljena i lokalni kifozačni ugao. Na kraju su izračunati broj fraktura nakon jedne godine i troškovi lečenja.

**Rezultati:**

Dve grupe nisu pokazale značajne razlike u bolu, pokretljivosti lumbalne kičme, visini prednje strane pršljena i lokalnom kifozačnom uglu nakon lečenja (P>0,050). Gustina minerala u kostima (BMD) bila je viša u grupi lečenoj denosumabom (DEN) na 6 i 12 meseci nakon lečenja, dok je b izomer C-terminalnog telopetida tipa I kolagena (b-CTX) bio ni'i (P<0,050). Nije utvrđena statistički značajna razlika između grupa u broju preloma nakon jedne godine (P>0,050), dok su ukupni troškovi lečenja bili niži u grupi lečenoj denosumabom u poređenju sa grupom lečenom zoledronskom kiselinom (P<0,050).

**Zaključak:**

DEN i ZOL efikasno poboljšavaju prognozu pacijenata sa osteoporotskim kompresionim frakturama pršljenova (OVCF) nakon PKP, ali DEN može značajnije poboljšati gustinu minerala u kostima (BMD) i metabolizam kostiju uz veše ekonomske koristi.

## Introduction

Osteoporosis (OP) is an age-related metabolic bone disease whose incidence increases with age [1]. Statistics show that in 2016, the incidence of OP in the global population over 60 years old was above 36% [2]. As of 2022, the global cumulative number of OP cases has exceeded 200 million, and the World Health Organization predicts a rising prevalence of OP in the coming decades [3]. Patients with OP are highly susceptible to fractures due to bone loss, bone microstructure destruction, and increased bone brittleness, among which osteoporotic vertebral compression fractures (OVCF) are the most common, with over 40% of OP patients experiencing OVCF [4]. OVCF can cause severe pain and difficulty in turning over while affecting the operation of cardiopulmonary function due to spinal compression [5]. In clinical practice, percutaneous kyphoplasty (PKP), a repeatedly validated procedure with its effectiveness and safety, has been advocated for OVCF [6] [7]. Following PKP, the administration of bone resorption drugs or osteogenic agents is typically required to enhance patient prognosis and facilitate rehabilitation.

Among them, Denosumab (DEN) is an inhibitor of the receptor activator of nuclear factor kappa B ligand (RANKL) – the main medium for osteoclast differentiation, activation and survival. DEN can interfere with the binding of RANKL to its receptors, inhibit osteoclast formation, and balance bone absorption and bone formation [8]. While Zoledronic acid (ZOL) is a bisphosphonate with the strongest pharmacological activity, which exerts anti-bone reabsorption by inhibiting farnesyl diphosphate synthases [9]. Both DEN and ZOL have achieved excellent results in treating OVCF patients after PKP [10] [11]. However, few studies have compared their advantages and disadvantages in treating OVCF, so their application choice remains controversial.

Hence, this study compares the clinical efficacy of DEN and ZOL treatments for OVCF, offering insights and guidance for future clinical OVCF management.

## Materials and methods

### Participants

A total of 167 patients who had OVCF and underwent PKP at our hospital between June 2020 and August 2023 were selected as the research participants. These patients met the inclusion criteria, which established that they were diagnosed with OVCF through imaging examinations [12], received PKP surgery at our institution [13], and consented to be part of this study with complete medical records. Subsequently, 128 patients were included after excluding individuals who had pathological fractures due to malignancies or metabolic osteopathy, drug-induced osteoporosis resulting from prolonged hormone use, vertebral burst fractures associated with spinal cord injury and nerve compression, previous vertebral compression fractures, fractures in other areas, multi-level thoracolumbar compression fractures, or those medically unfit for surgery due to age or underlying health conditions. Then, using a random number table, they were divided into a DEN group (n=64) and a ZOL group (n=64) for DEN and ZOL therapy, respectively. This study has obtained approval from our hospital’s ethics committee and informed consent from all participants (No.23-city-21).

### Surgical plan

After admission, all patients were given symptomatic treatment such as analgesia, fluid replacement, and postural reduction. The same surgical team performed PKP after a comprehensive preoperative routine examination. The patient is placed on the operating table and equipped with monitoring equipment to track vital signs. Local anesthesia, along with intravenous sedation, is typically administered to ensure the comfort and safety of the patient during the procedure. The surgeon utilizes X-ray guidance (Nanjing Puai Medical Equipment Co., Ltd., PLX112C) to direct specialized puncture needles through the skin and soft tissues into the affected vertebrae. Usually crafted from rigid materials, these needles offer precise control and guidance. Once the needles are inserted into the fractured vertebrae, the surgeon utilizes a special expander to pre-expand them, ensuring ample and stable space for injecting bone cement. Following the pre-dilatation procedure, a molded balloon (typically composed of polyethylene or polyester) is inserted through the puncture needle and positioned within the vertebral body. The vertebral body within the compressed fracture segment is realigned by inflating the balloon. Subsequently, bone cement injection is performed, wherein polymethylmethacrylate (PMMA) is injected into the balloon via a puncture needle. This injection process enables the bone cement to fill the cavity, solidify, and stabilize the compressed fractured vertebrae. Postoperatively, all patients were managed according to our department’s painless ward management model and were given ECG monitoring, routine fluid rehydration, and other treatment programs. In addition, routine X-rays and plain CT scans were performed to check for any leakage of bone cement. All patients were followed up once a month for one year.

### Treatments

After the operation, the DEN group was given subcutaneous injections of DEN (60 mg) twice a year. While patients in the ZOL group were intravenously infused with 5 mg of ZOL once a year. All patients were supplemented with vitamin D3 (125 IU) and calcium carbonate (600 mg) daily during the treatment. Both groups were treated continuously for one year.

### Endpoints

(1) Surgical results and postoperative adverse reactions were analyzed. (2) Bone mineral density (BMD) of the lumbar spine L1-L4 was detected by X-ray BMD meter before surgery and 6 months and 12 months after treatment. In addition, the patient’s fasting cubital vein blood was collected for enzyme-linked immunosorbent assays (ELISAs) of bone alkaline phosphatase (BAP), total procollagen type 1 aminoterminal propeptide (TP1NP), bone gamma carboxyglutamic acid containing proteins (BGP), and β isomer of C-terminal telopeptide of type I collagen (β-CTX). Besides, pain assessment was made using the Visual Analogue Scale (VAS; on a 10-point scale, higher scores suggest more obvious pain) [14], and lumbar mobility evaluation was performed with the Japanese Orthopaedic Association (JOA) score (score range: 0–29 points; higher scores indicate better lumbar mobility) [15]. Moreover, the anterior vertebral height (the distance between the upper and lower laminae of the anterior margin of the injured vertebra) and local kyphotic angle (i.e. the angle between the extension along the endplate of the fractured vertebral body and the intersection of the two) were measured. (3) The one-year re-fracture rate and the total treatment cost were counted.

### Statistical methods

This study imported data into SPSS24.0 for statistical analysis, with P<0.050 as the threshold of statistical significance. Categorical variables were presented in the form of (n (%)), and the chi-square test was used for inter-group comparisons. Continuous variables, described as (x̄±s), were compared between groups using the independent sample t-test and among multiple groups using one-way analysis of variance (ANOVA) plus least significant difference (LSD) intra-group test.

## Results

### The two groups were not obviously different in clinical data

To ensure the reliability of the research results, we first compared the baseline data of patients, such as age, sex, duration of OP, and fracture site. We found no significant inter-group difference (all P>0.050, Table 1).

**Table 1:** Clinical baseline information.

Group	Male	Female	Age	Duration of OP<br>(years)	Lumbar fracture	Thoracic fracture
DEN (n=64)	26 (40.63)	38 (59.38)	68.59±8.16	7.44±3.45	34 (53.13)	30 (46.88)
ZOL (n=64)	22 (34.38)	42 (65.63)	66.83±5.56	7.61±2.76	38 (59.38)	26 (40.63)
χ^2^ (t)	0.533	1.431	0.311	0.508		
P	0.465	0.155	0.756	0.476		

### Surgical conditions

All patients successfully completed the operation, with no vascular or nerve injury during the operation or postoperative infections. Adverse reactions occurred in 12.50% of patients in the DEN group and 15.63% in the ZOL group, with no significant intergroup difference in the total incidence of postoperative adverse reactions (P=0.611, Table 2).

**Table 2:** Postoperative adverse effects.

Group	Lower back pain	Chest tightness	Cement leakage	Cement rejection	Lower extremity<br>numbness	Total incidence
DEN (n=64)	3 (4.69)	2 (3.13)	1 (1.56)	0 (0.0)	2 (3.13)	8 (12.50)
ZOL (n=64)	3 (4.69)	3 (4.69)	1 (1.56)	1 (1.56)	2 (3.13)	10 (15.63)
χ^2^						0.259
P						0.611

### The DEN group had a better prognosis than the ZOL group

**Figure 1:**
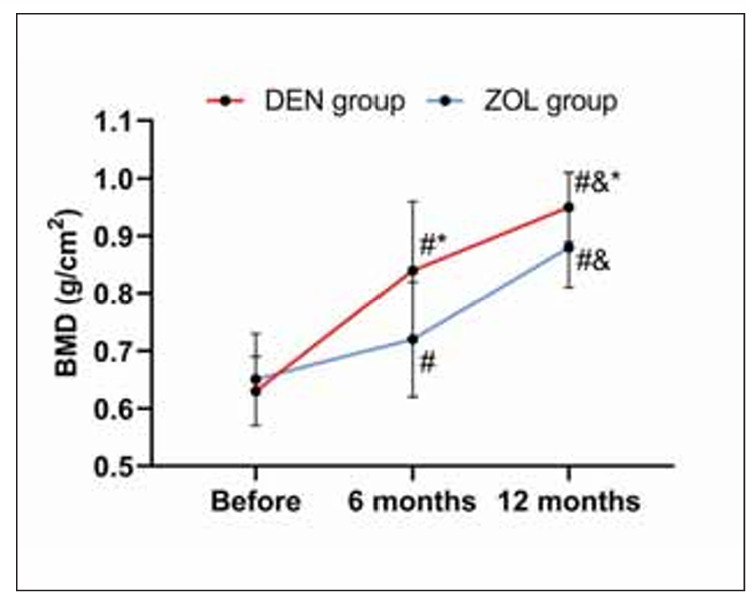
Comparison of BMD. Note: vs before treatment #P<0.050, vs 6 months after treatment &P<0.050, vs ZOL group *P<0.050.

The two groups showed no evident difference in BMD before treatment (P=0.112). BMD in both groups increased after 6 months of treatment, with a higher level in the DEN group versus the ZOL group (P<0.001). After 12 months of treatment, the BMD of both groups increased further, especially in the DEN group (P>0.001, Figure 1).

### DEN group showed lower prognostic bone metabolism than the ZOL group

**Figure 2:**
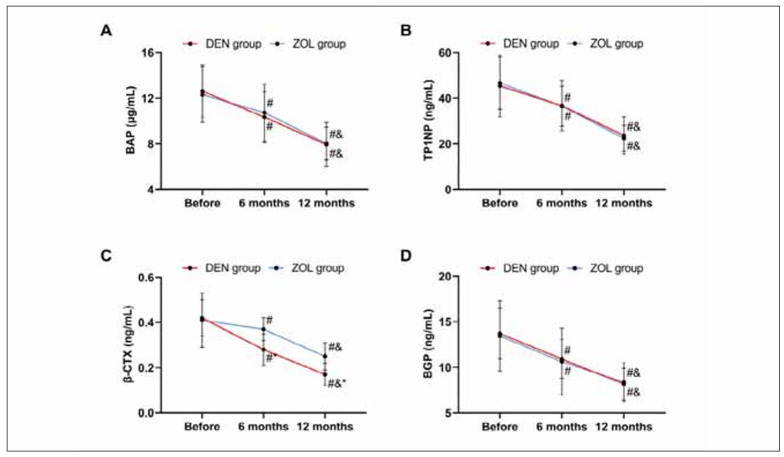
Comparison of bone metabolism. A: comparison of BAP, B: comparison of TP1NP, C: comparison of β-CTX, D: comparison of BGP. Note: vs before treatment #P<0.050, vs 6 months after treatment &P<0.050, vs ZOL group *P<0.050.

Similarly, no statistical significance was found in the results of bone metabolism indexes between the two groups before treatment (all P>0.050). After 6 months and 12 months of treatment, BAP, TP1NP, and BGP in the DEN group were not different from those in the ZOL group (all P>0.050), but β-CTX was lower (P<0.050). The bone metabolism indexes of both groups decreased after treatment and reached the lowest values at 12 months (all P<0.050, Figure 2).

### There was no difference between the two groups in pain and lumbar mobility

**Figure 3:**
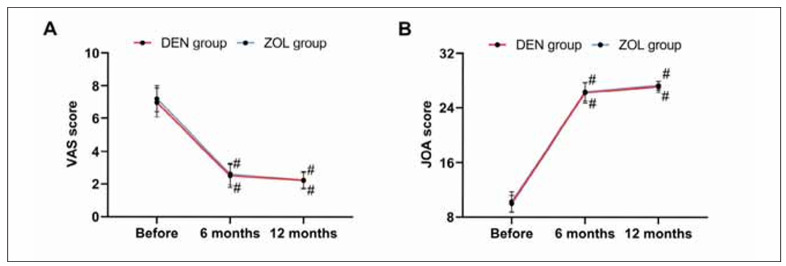
Comparison of pain and lumbar mobility. A: comparison of VAS, B: comparison of JOA. Note: vs before treatment #P<0.050, vs 6 months after treatment &P<0.050.

No evident differences were found in the scores of VAS and JOA between the two groups before surgery and 6 and 12 months after treatment (all P>0.050). The VAS score decreased after treatment in both groups, while the JOA score increased (all P<0.050, Figure 3).

### There was no difference in vertebral conditions between the two groups

**Figure 4:**
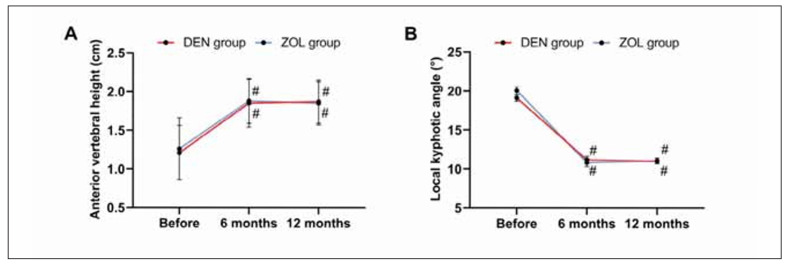
Comparison of vertebral conditions. A: comparison of anterior vertebral height; B: comparison of local kyphotic angle. Note: vs before treatment #P<0.050, vs 6 months after treatment & P<0.050.

The two groups were not statistically different in anterior vertebral height and kyphotic angle before and after treatment (all P>0.050). In both groups, the anterior vertebral height increased after 6 and 12 months of treatment, and the kyphotic angle decreased (all P<0.050, Figure 4).

### The two groups showed no marked difference in the recurrence rate

The one-year re-fracture rate was 3.13% in the DEN group and 4.69% in the ZOL group, showing no significant difference in the prognostic re-fracture rate (P=0.648, Table 3).

**Table 3:** Prognostic 1-year re-fracture rate.

Group	Re-fracture	No-fracture
DEN (n=64)	2 (3.13)	62 (96.88)
ZOL (n=64)	3 (4.69)	61 (95.31)
χ^2^	0.208	
P	0.648	

### Comparison of economic benefits

**Figure 5:**
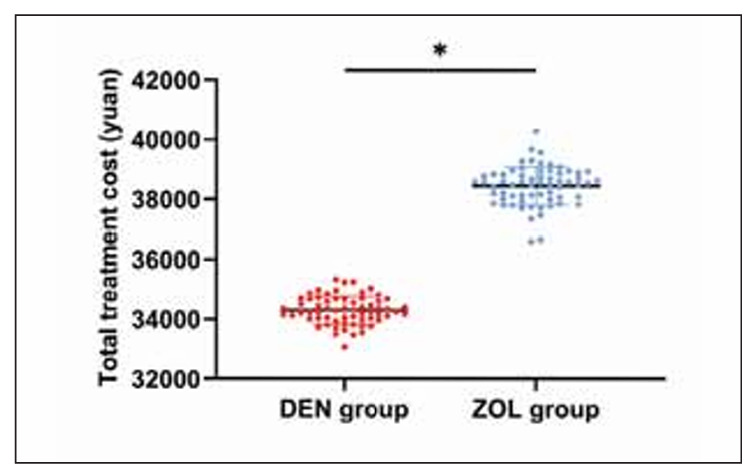
Comparison of economic benefits. Note: *P<0.001.

Finally, by analyzing the treatment costs of the two groups, it was found that the treatment cost of the DEN group was (34303.42±465.84) yuan, while that of the ZOL group was (38446.59±644.68) yuan. Compared with the ZOL group, the treatment cost of the DEN group was lower (P<0.001, Figure 5).

## Discussion

In this study, we observed that DEN has a more significant positive impact on bone metabolism and BMD in patients with OVCF compared to ZOL, indicating its higher clinical utility.

When comparing BMD and bone metabolism, it was found that DEN led to higher BMD (BMD at 6 and 12 months of treatment in the DEN group was 0.84±0.12 g/cm^2^ and 0.84±0.12 g/cm^2^, BMD at 6 and 12 months of treatment in the ZOL group was 0.72±0.10 g/cm^2^ and 0.88±0.07 g/cm^2^, all P<0.001) and lower β-CTX (β-CTX at 6 and 12 months of treatment in the DEN group was 0.28±0.07 ng/mL and 0.17±0.05 ng/mL, β-CTX at 6 and 12 months of treatment in the ZOL group was 0.37±0.05 ng/mL and 0.25±0.06 ng/mL, all P<0.001) after 6 and 12 months of treatment, indicating that DEN has a better effect on improving BMD and bone metabolism in OVCF patients. As a third-generation bisphosphonate, the mechanism of ZOL is to replace P-O-P in the pyrophosphate structure that is easily hydrolyzed by enzymes in vivo with P-C-P, synthesizing stable compounds that are not easily hydrolyzed by enzymes in the body [16]. The drug plays an anti-bone reabsorption role by inhibiting osteoclast function and reducing the osteoclast count, thus lowering the risk of osteoporotic fractures [17]. DEN can directly inhibit the formation and function of osteoclasts by inhibiting the binding of RANKL to its receptors, thereby reducing bone resorption [18]. As is well known, β-CTX is one of the downstream synthetic products of RANKL [19]. Therefore, DEN has a more direct and rapid inhibitory effect on β-CTX. Meanwhile, since inhibiting ZOL to osteoclasts requires binding to bone minerals, the strong affinity for hydroxyapatite may limit the uniform distribution of ZOL in bone [20]. DEN, on the other hand, is a circulating antibody that reaches all parts of the bone, especially deep in the bone [21], and thus may have a stronger inhibitory effect on bone remodelling than ZOL. This may also be one of the reasons why the improvement of BMD and bone metabolism in the ZOL group was not as significant as that in the DEN group. In the comparison of pain (DEN group were 6.97±0.89, 2.50±0.71, 2.23±0.53 before treatment, 6 months and 12 months of treatment, respectively, ZOL group were 7.20±0.80, 2.61±0.66, 2.23±0.50 before treatment, 6 months and 12 months of treatment, respectively, all P>0.050) and lumbar spine mobility (DEN group were 9.92±1.21, 26.23±1.54, 27.13±0.83 before treatment, 6 months and 12 months of treatment, respectively, ZOL group were 10.23±1.47, 26.36±1.38, 27.30±0.66 before treatment, 6 months and 12 months of treatment, respectively, all P>0.050) before and after treatment and the re-fracture rate (DEN group: 3.13%, ZOL group: 4.69%, P=0.648), we found no significant difference between DEN and ZOL, suggesting that both drugs have ideal and stable effects on lumbar rehabilitation in patients with OVCF, which is consistent with many research results [22] [23] and highlights the excellent application value of the two in the treatment of OVCF. Therefore, we still cannot completely ignore other advantages of ZOL, such as convenient intravenous administration and high bioavailability, especially for patients with severe gastrointestinal intolerance or poor oral absorption. In addition, it has a long half-life and can be used once a year, improving patient compliance. However, when using ZOL, we should also note that ZOL is only applicable to patients with primary OP, creatinine clearance 35 mL/min, and no abnormalities in blood calcium. In addition, proper hydration should be performed before and after medication to reduce the occurrence of adverse reactions. Approximately 39% of ZOL is excreted from the kidneys in its original form, requiring the patient to have good renal function. Moreover, blood calcium may fluctuate after ZOL use, so calcium and vitamin D supplementation are required after medication to prevent hypocalcemia.

On the other hand, when comparing the economic impact, the treatment cost in the DEN group was significantly lower at 34,303.42±465.84 yuan compared to the ZOL group at 38,446.59±644.68 yuan (P<0.001). This variance is primarily attributed to DEN’s lower market cost than ZOL. However, the results may also be underrepresented because China’s health system allows patients to use citizen health insurance to cover most of the cost of treatment. So, the differences in the economic effects of DEN versus ZOL for OVCF need to be comprehensively evaluated using case reports from more countries.

However, this study’s small number of cases may have contributed to the chance of results. At the same time, DEN and ZOL are both drugs that inhibit osteoclast activity. Still, this paper does not discuss the therapeutic effect of drugs that promote osteoclast activity (such as teriparatide). This is due to the high cost of such drugs, the greater economic burden on patients, and the need to inject once a day, making it difficult to ensure long-term follow-up. In the future, we also need to complement the advantages and disadvantages of different drugs in OVCF further in conjunction with other studies.

In combination, both DEN and ZOL exhibit a consistent, safe, and efficacious capability in enhancing outcomes for patients with OVCF following PKP, leading to effective pain relief and enhancement of lumbar function recovery. Particularly, DEN demonstrates superior efficacy in enhancing BMD and bone metabolism in OVCF patients, offering cost-effective treatment and notable economic advantages, thus warranting its recommendation as the preferred option for clinical application.

## Dodatak

### Informed consent

The established study protocol (Protocol Approval number L20021, dated June 2020. ) was approved by the human ethics committee of Jinan Second People’s Hospital. The study adheres to the laws of China and the 2008 version of the Declaration of Helsinki.

### Funding

Not applicable.

### Availability of data and materials

The data supporting this study’s findings are available from the corresponding author upon reasonable request.

### Author contributions

Jiayin Meng designed the study, Shouda Gao wrote the manuscript, Lijun Zhang collected and analyzed data, and Liyan Liu revised the manuscript. Shouda Gao and Lijun Zhang contributed equally to this work and are co-first authors. All authors read and approved the final submitted manuscript.

### Conflict of interest statement

All the authors declare that they have no conflict of interest in this work.
